# Hydrogen production coupled with water and organic oxidation based on layered double hydroxides

**DOI:** 10.1002/EXP.20210050

**Published:** 2021-12-16

**Authors:** Yingjie Song, Kaiyue Ji, Haohong Duan, Mingfei Shao

**Affiliations:** ^1^ State Key Laboratory of Chemical Resource Engineering Beijing University of Chemical Technology Beijing P. R. China; ^2^ Department of Chemistry Tsinghua University Beijing P. R. China

**Keywords:** hydrogen production, layered double hydroxides, organic oxidation, oxygen evolution reaction, water splitting

## Abstract

Hydrogen production via electrochemical water splitting is one of the most green and promising ways to produce clean energy and address resource crisis, but still suffers from low efficiency and high cost mainly due to the sluggish oxygen evolution reaction (OER) process. Alternatively, electrochemical hydrogen‐evolution coupled with alternative oxidation (EHCO) has been proposed as a considerable strategy to improve hydrogen production efficiency combined with the production of high value‐added chemicals. Although with these merits, high‐efficient electrocatalysts are always needed in practical operation. Typically, layered double hydroxides (LDHs) have been developed as a large class of advanced electrocatalysts toward both OER and EHCO with high efficiency and stability. In this review, we have summarized the latest progress of hydrogen production from the perspectives of designing efficient LDHs‐based electrocatalysts for OER and EHCO. Particularly, the influence of structure design and component regulation on the efficiency of their electrocatalytic process have been discussed in detail. Finally, we look forward to the challenges in the field of hydrogen production via electrochemical water splitting coupled with organic oxidation, such as the mechanism, selected oxidation as well as system design, hoping to provide certain inspiration for the development of low‐cost hydrogen production technology.

## Graphical abstract

In this review, we have summarized the latest research progress of hydrogen production from the perspectives of designing efficient LDHs‐based electrocatalysts for OER and EHCO. Meanwhile, the challenges in the field of hydrogen production via electrochemical water splitting coupled with organic oxidation have been raised.

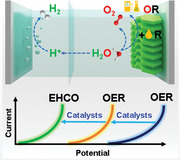

## 1 INTRODUCTION

Hydrogen has been regarded as the most potential and practical energy carrier due to its virtue of high energy density and zero greenhouse gas emissions after its depletion.^[^ ^1^, ^2^, ^3^ ^]^ The vigorous development and promotion of hydrogen energy can effectively reduce carbon emissions, which is of great importance for achieving the sustainable development of human society. Traditional techniques of hydrogen production involving chemicals cracking and fossil fuel reforming are low cost and easily scalable, but CO_2_ and pollutant by‐products are always inevitable.^[^ ^4^, ^5^, ^6^ ^]^ In contrast, hydrogen production through electrochemical water splitting, namely using water molecules as hydrogen source and electricity as the power to obtain high purity hydrogen products, avoids the aforementioned pollutant to a large extent and achieves zero carbon emission.^[^ ^7^, ^8^, ^9^ ^]^ However, the high cost brought by extensive energy consumption remains as the bottleneck that restricts its application.

**FIGURE 1:**
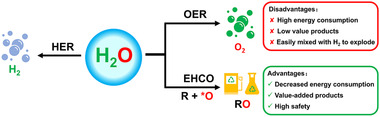
Comparison between OER involved electrochemical hydrogen production and EHCO involved electrochemical hydrogen production

In general water splitting cell, hydrogen evolution reaction (HER) at cathode is coupled with the oxygen evolution reaction (OER) at anode. Compared with HER, OER is always sluggish due to a complex four‐electron transfer process, which leads to a high voltage to drive the efficient hydrogen production.^[^ ^10^, ^11^, ^12^, ^13^ ^]^ Despite the efforts made on the development of effective OER catalysts to lower the overpotential, the energy consumption of electrochemical splitting water is still high. Meanwhile, the final O_2_ product of OER is less‐valued and dangerous when it mixes with simultaneously produced H_2_ in the electrolyzer.^[^ ^13^, ^14^ ^]^ In contrast, coupling HER with thermodynamically more favorable small molecules oxidation reaction instead of OER cannot only effectively reduce overpotential of the anode reaction, but also bring higher economic benefits by the high value‐added products obtained from oxidation (Figure 1). Inspired by this idea, more and more works are focusing on electrochemical hydrogen‐evolution coupled with alternative oxidation (EHCO) in recent years.

**FIGURE 2:**
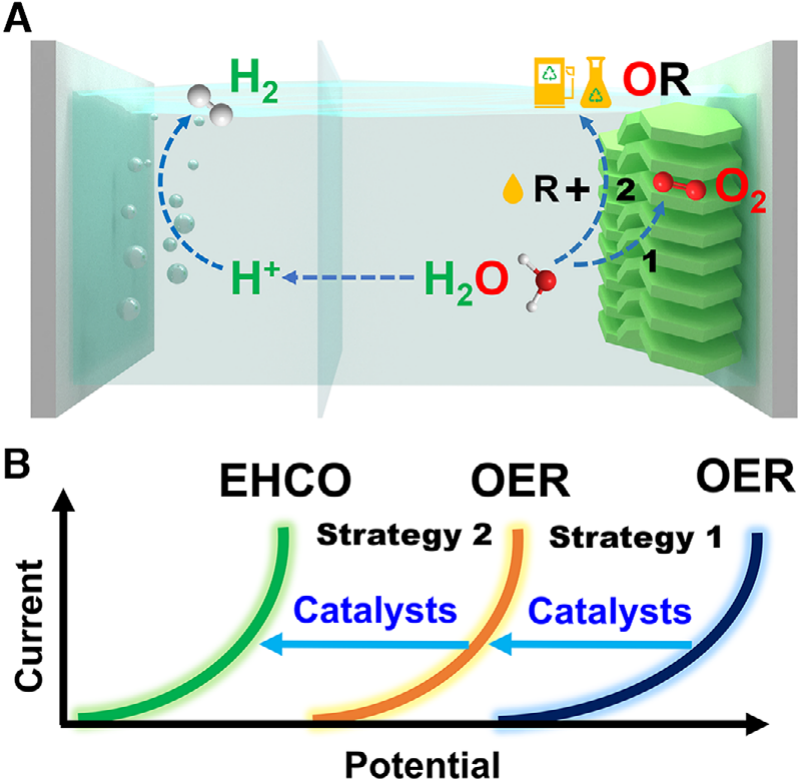
(A) Schematic illustration of two strategies on reducing the cost and improving the economic benefits of electrochemical water splitting. (B) Comparison of two strategies: Enhanced OER and EHCO

To reduce the energy consumption and oxidation efficiency for OER and EHCO, the development of efficient electrocatalysts with low cost is of vital significance. Layered double hydroxides (LDHs) are typical widely reported layered materials, which are composed of the positively charged host layers and the interlayer charge balancing anions. The flexibility of composition and structure for LDHs significantly expand their application in the field of electrochemical energy storage and conversion, especially as advanced electrocatalysts.^[^ ^15^, ^16^, ^17^, ^18^, ^19^ ^]^ Moreover, a variety of derived electrocatalysts have also been developed by using LDHs as precursors through topochemical conversions, such as oxides, phosphides, sulfides, and alloy catalysts.^[^ ^20^, ^21^, ^22^, ^23^, ^24^ ^]^ In this review, we summarized the latest research progress of hydrogen production in terms of the design of LDHs‐based efficient electrocatalysts for both OER and EHCO (Figure 2A). In particular, we discussed the function of the composition and structure of LDHs materials on their electrocatalytic performance in detail. The applications of different LDHs electrocatalysts in various oxidation reactions (such as OER, alcohol oxidation, and furfural compounds oxidation) are also presented. Moreover, remaining challenges and prospect from the perspectives of mechanism investigation, reaction efficiency, and device design are also summarized and proposed. We hope this review can give inspiration to the design of advanced systems and efficient catalysts to promote the development of electrochemical hydrogen production.

## 2 HYDROGEN PRODUCTION COUPLED WITH OER BASED ON LDHS

The anodic OER in water splitting is a complicated and sluggish four‐electron transfer process, giving rise to a large overpotential to overcome the energy barrier for driving water splitting. To meet this challenge, kinds of electrocatalysts with various compositions (e.g., sulfide, phosphide, oxide, hydroxide) and structures (e.g., nanosheets array, heterojunction) were designed and reported.^[^ ^25^, ^26^, ^27^, ^28^ ^]^ Among them, LDHs, especially NiFe‐LDH, display superior catalytic performance for OER in alkaline media than most of the transition metal catalysts and several noble metal oxides electrocatalysts such as RuO_2_ or IrO_2_.^[^ ^29^, ^30^, ^31^, ^32^, ^33^, ^34^ ^]^ Moreover, a variety of functionalized electrocatalysts derived from LDHs could realize a higher catalytic activity and stability to meet the stand of scale‐up hydrogen production. In this section, we concluded the latest progress of LDH‐based catalysts for highly enhanced OER (Table 1) and discussed the design and regulation of LDHs‐based OER catalysts in terms of the underlying principles.

**TABLE 1:** OER performances based on LDHs electrocatalysts

Modification strategy	Catalyst	Electrolyte	Overpotential @ current density (mV @ mA cm^−2^)	Tafel slope (mV dec^−1^)	Durability (hours @ mA cm^−2^)	Ref.
Hierarchical nanostructure construction	NiFe LDHs/NF	1 M KOH	240 @ 10	−	−	^[^ ^15^ ^]^
	NiFe‐LDH	1 M KOH	224 @ 10	52.8	10 @ 10	^[^ ^31^ ^]^
	NiFe‐LDH HMS	1 M KOH	239 @ 10	53	−	^[^ ^32^ ^]^
	NiFe‐LDH DSNCs	1 M KOH	246 @ 20	71	20 @ 50	^[^ ^34^ ^]^
	NiFe‐LDH SSNCs	1 M KOH	261 @ 20	117	−	
Cation doping/anion intercalation	NiFeMn‐LDH	1 M KOH	289 @ 20	47	10 @ 15	^[^ ^41^ ^]^
	NiFeV‐LDH	1 M KOH	195 @ 20	42	22 @ 18	^[^ ^42^ ^]^
	NiFeV‐LDH	1 M KOH	287 @ 10	53.7	−	^[^ ^43^ ^]^
	NiFeTi‐LDH	1 M KOH	307 @ 10	58.3	−	
	NiFeCr‐LDH	1 M KOH	295 @ 10	67	−	
	NiFeMn‐LDH	1 M KOH	313 @ 10	49.3	−	
	NiFeCo‐LDH	1 M KOH	290 @ 10	78.1	−	
	NiFeCu‐LDH	1 M KOH	317 @ 10	86.9	−	
	NiFeAl‐LDH	1 M KOH	374 @ 10	59.6	−	
	NiFeRu‐LDH	1 M KOH	29 @ 10	31	10 @ 10	^[^ ^44^ ^]^
	MoO_4_^−^/NiFe‐LDHs	1 M KOH	280 @ 10	40	50 @ 10	^[^ ^45^ ^]^
	PO_4_^3−^/NiFe‐LDH	1 M KOH	260 @ 10	42.1	−	^[^ ^46^ ^]^
	HPO_3_^2−^/NiFe‐LDH	1 M KOH	270 @ 10	40.6	−	
	H_2_PO_2_^3−^/NiFe‐LDH	1 M KOH	240 @ 10	37.7	−	
Exfoliation	NiFe‐LDH	1 M KOH	300 @ 10	40	13 @ 10	^[^ ^53^ ^]^
	NiCo‐LDH	1 M KOH	330 @ 10	41	13 @ 10	
	CoCo‐LDH	1 M KOH	350 @ 10	45	13 @ 10	
	NiCo‐LDH	1 M KOH	367 @ 10	40	6 @ 10	^[^ ^54^ ^]^
	CoAl‐LDH	1 M KOH	340 @ 10	70.5	110 @ 10	^[^ ^59^ ^]^
Vacancies/defects construction	CoFe‐LDH‐Ar	1 M KOH	266 @ 10	37.6	−	^[^ ^56^ ^]^
	H_2_O‐plasma exfoliated CoFe‐LDH	1 M KOH	290 @ 10	36	−	^[^ ^57^ ^]^
	PM‐LDH	1 M KOH	230 @ 10	47	10 @ 100	^[^ ^60^ ^]^
	CoGa‐LDH/SSM	1 M KOH	258 @ 10	34.1	10 @ 70	^[^ ^61^ ^]^
	NiFe‐LDH‐V_Fe_	1 M KOH	245 @ 10	70	−	^[^ ^62^ ^]^
	NiFe‐LDH‐V_Ni_	1 M KOH	229 @ 10	62.9	−	
LDHs‐based composites	CoNiP@LDHs	1 M KOH	216 @ 10	45	50 @ 500	^[^ ^71^ ^]^
	^s^Au/NiFe‐LDH	1 M KOH	237 @ 10	36	100 @ 20	^[^ ^72^ ^]^
	NiFe‐LDH/GO	1 M KOH	206 @ 10	39	20 @ 8	^[^ ^73^ ^]^
	NiFe‐LDH/ CNTs	1 M KOH	250 @ 5	31	5 @ 1	^[^ ^29^ ^]^
	NiFe‐LDH NS@DG	1 M KOH	210 @ 10	52	10 @ 10	^[^ ^74^ ^]^
	CQD/NiFe‐LDH	1 M KOH	235 @ 10	30	2.5 @ 1	^[^ ^75^ ^]^
	Cu@NiFe‐LDH	1 M KOH	199 @ 10	27.8	100 @ 48	^[^ ^76^ ^]^
	Ag NW@NiMn‐LDH	1 M KOH	270 @ 10	40.2	10 @ 28	^[^ ^77^ ^]^
	NiFe‐LDH‐NiSe	1 M KOH	240 @ 100	65.6	50 @ 12	^[^ ^78^ ^]^
	NiCoP@NiMn‐LDH	1 M KOH	293 @ 100	43.7	50 @ 100	^[^ ^79^ ^]^

One of the best‐known advantages of LDHs is the tunability of composition for both layer cation and interlayer anion, which greatly broaden their application. Therefore, a series of works surrounding the controllable preparation, structural modulation, and performance enhancement of LDHs‐based catalysts have been reported.^[^ ^35^, ^36^, ^37^, ^38^, ^39^ ^]^ It is worth mentioning that our group firstly synthesized Fe containing‐LDHs via a coprecipitation strategy and found their excellent electrocatalytic properties towards OER and the oxidation reaction of organic molecules.^[^ ^40^ ^]^ Afterward, NiFe‐LDH has been reported to display superior OER performance and has been recognized as one the best OER catalysts till now. To further enhance the OER activity of LDHs, the doping strategy of third metal ions (e.g., Co, Mn, V, etc.) has been developed.^[^ ^41^, ^42^, ^43^, ^44^ ^]^ For instance, the OER activity of NiFe‐LDH can be significantly promoted by cation doping due to the effective electron transfer from Fe^3+^ in NiFe‐LDH to the doping cation (e.g., Co^2+^, V^3+^, and Cr^3+^), which facilitates the adsorption of oxygen species.^[^ ^43^ ^]^ Meanwhile, the interlayer anion also plays a crucial role in the OER performance of LDHs probably owing to the following reasons: First, the size of interlayer anion determines the interlayer distance, which thereby affects the electrochemically active surface area and the diffusion of reactants and products. Secondly, suitable interlayer anions may serve as additional active sites or enhance the activity of laminate metal active sites.^[^ ^45^, ^46^ ^]^ For instance, H_2_PO_2_^−^ intercalated NiFe‐LDH exhibited better OER performance due to the regulation of the electronic structure of Ni sites, which resulted in an optimized kinetic process for OER.^[^ ^46^ ^]^

To better understand the OER mechanism and design of LDHs catalysts, recent efforts have been made to demonstrate the activity origin and true active sites of LDHs.^[^ ^47^, ^48^, ^49^, ^50^, ^51^ ^]^ For instance, we found out that metal active sites can be *in‐situ* activated through the formation of hydrogen vacancies under the OER condition.^[^ ^47^ ^]^ Moreover, our recent work proved that the edge sites of LDHs are more active than the inner plane sites.^[^ ^48^ ^]^ This inspired us to construct LDHs catalysts with more exposed edge sites or enhance the intrinsic activity of plane sites of LDHs. Accordingly, the rational design of LDHs with hierarchical nanostructure has been widely reported for boosting OER performances. Among them, the construction of hollow nanostructures has displayed distinct advantages, such as large surface area, multiple interfaces, more exposed edge sites, and optimized diffusion process. Our group reported hierarchical NiFe‐LDH hollow microspheres with a small onset overpotential (239 mV at 10 mA cm^−2^) for OER, a remarkably low Tafel slope (53 mV dec^−1^) as well as robust durability.^[^ ^29^ ^]^ Besides, Growing LDHs nanosheets directly on the surface of conductive substrate to form a 3D architectural nanosheets array is widely used in the fabrication of OER electrode with more exposed edge sites.^[^ ^31^, ^33^, ^42^, ^52^ ^]^ We first demonstrated a facile and economic electrochemical strategy to obtain a variety of Fe‐containing LDHs nanoarrays for efficient OER.^[^ ^31^ ^]^ Specifically, the as‐prepared NiFe‐LDH nanoarrays exhibit excellent OER activity with a low overpotential (0.224 V at 10.0 mA cm^−2^) and robust stability for over 50 h, which is one of the NiFe‐LDH catalyst with the best OER performance reported so far. All these reports show that the optimization in micro/nanostructure design for LDHs is not only a guarantee of excellent performance on OER, but also laid foundation for practical applications in hydrogen production.

Practically, the lamellas of LDHs are easy to stack together in the fabrication process, which prevents the efficient contact of active sites with reactants and thereby greatly limits the OER activity of LDHs. To overcome this issue, plenty of strategies were developed to exfoliate bulk LDH sheets into ultrathin nanosheets with larger surface area and higher exposure of active centers, such as liquid exfoliation by using organic solvent,^[^ ^17^, ^53^, ^54^, ^55^ ^]^ and dry exfoliation methods.^[^ ^56^, ^57^, ^58^ ^]^ In practical application, the activity and durability of these exfoliated LDHs will be greatly compromised when using binders to coat these materials on the electrode. Therefore, the development of *in‐situ* exfoliation strategy of LDHs nanosheets array on current collector is necessary. In our recent report, we develop a fabrication strategy of ultrathin CoAl‐LDH nanosheets array via an *in‐situ* exfoliation strategy.^[^ ^59^ ^]^ Through this strategy, CoAl‐LDH nanosheets array was exfoliated on the conductive substrate and the ultra‐thin structure was obtained with the thickness of only ∼2.93 nm.

The construction of vacancies/defects is a common method to regulate the electronic structure of pristine nanomaterials and break the limits of activity for further application. Thus, a number of previous reports have been investigated that the vacancies construction on LDHs could activate the plane sites and enhance the intrinsic activity of LDHs.^[^ ^56^, ^57^, ^60^, ^61^, ^62^ ^]^ However, the characterization vacancies structure is usually *ex‐situ*, and the reconstruction of LDHs under OER environment is quite common.^[^ ^63^, ^64^ ^]^ Therefore, it is important to pay more attention to the *in‐situ* changes of vacancies structure under the reaction conditions in order to inspire the researchers to design advanced catalysts rationally.

Furthermore, various electrocatalysts for OER have been obtained by using LDHs as precursors through topochemical conversions, such as transition metal phosphides, sulfides, and alloy catalysts.^[^ ^65^, ^66^, ^67^ ^]^ Our group reported a 2D ultrathin Fe doped CoP nanosheets array via *in‐situ* transformation of CoFe‐LDH for efficient OER with an onset potential of 1.48 V *vs*. RHE. The excellent OER activity of CoFeP can be ascribed to the optimized electronic structure of Co species, which heightened the adsorption of H_2_O molecule on catalyst surface.^[^ ^65^ ^]^ Similarly, constructing metal alloy derived from LDHs for OER has also been investigated. For instance, a MoO*_x_* modified NiFe alloy electrode derived from NiFe‐LDHs has been reported to boost the OER performance with a low overpotential of 276 mV due to a high surface area and a tuned Ni electronic state.^[^ ^68^ ^]^ These electrocatalysts inherit the nanostructure of LDHs, which provides an excellent mass transfer for reactants. It is worth mentioning that some reports pointed out that these catalysts are thermodynamically less stable under the OER condition, especially under high potential in strong alkaline media, which can be easily transformed into metal oxide/hydroxide phases.^[^ ^69^, ^70^ ^]^

To further boost the OER performance of LDHs, it is considered to combine LDHs with functional components to expand their applications.^[^ ^71^, ^72^, ^73^, ^74^, ^75^, ^76^, ^77^, ^78^, ^79^ ^]^ Many recent reports introduced the decoration of highly reactive components (such as noble metal single atoms, quantum dots, and oxides particles) on LDH to enhance OER activity. These works focus on the interfacial interactions between LDHs and active components, which contribute to accelerated charge transport and more stable charge distribution around the active sites. Thereinto, Zhang and co‐workers reported a single Au atom decorated NiFe‐LDHs toward highly efficient OER. The decoration of Au single atom provided electrons to NiFe‐LDH and balanced the charge distribution, which contributed to a 6‐fold enhanced OER performance.^[^ ^72^ ^]^ On the other hand, LDHs can also be compounded with other HER‐active catalysts to compensate for the disadvantages of LDHs in HER and achieve excellent performance on overall water splitting. For example, the well‐organized CoNiP@NiFe‐LDH nanosheets array shows outstanding performance toward overall water splitting with a cell voltage 1.44 V to reach 10 mA cm^−2^.^[^ ^71^ ^]^ The synergetic effects of the CoNiP core and the NiFe‐LDH shell simultaneously promote the HER and OER processes.

## 3 HYDROGEN PRODUCTION COUPLED WITH EHCO BASED ON LDHS

**FIGURE 3:**
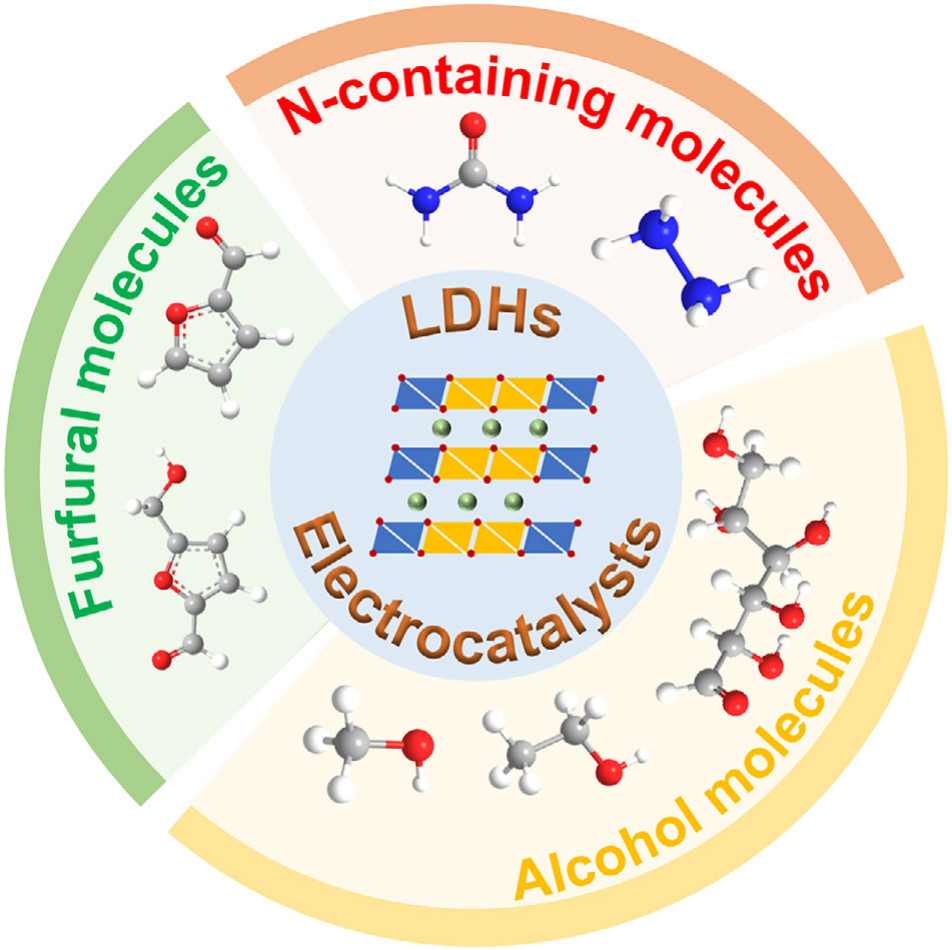
Schematic illustration of LDHs electrocatalysts for the oxidation of three major classes of organic molecules

Beyond the exploration of advanced electrocatalyst towards OER, another strategy is replacing the sluggish OER with thermodynamically more favorable molecules oxidation.^[^ ^80^, ^81^, ^82^, ^83^ ^]^ In this system, HER at cathode can be significantly accelerated due to the lower overpotential of anodic reactions (Figure 2B). Moreover, the value of anode products will be greatly increased compared to producing O_2_. Among them, the oxidation of three major classes of small molecules has been of most interest to researchers (Figure 3). The first category is the oxidation of various alcohols. In the early stages of research, the oxidation of some smaller molecular alcohols (e.g. methanol, ethanol) was of interest due to their application in direct fuel cells.^[^ ^84^ ^]^ Recently, the larger molecular alcohols (like glycerol and glucose) have been reported to be coupled with HER due to the diversity of products and their wide range of applications. Besides, the electrochemical oxidation of some furan compounds (especially furfural and 5‐hydroxymethyl furfural) is the hotspot of current research, which can be derived from biomass in nature and their numerous derivatives are commonly used as platform molecules and fuels in the chemical industry.^[^ ^85^ ^]^ The third major category is the oxidation of N‐containing molecules, such as urea and hydrazine. These small nitrogen‐containing molecules are widely available and possess lower theoretical oxidation potentials, which contributes to a lower overall reaction voltage than water splitting. In addition, these oxidation reactions are also strong candidates for direct fuel cell anode reactions.^[^ ^86^ ^]^ Interestingly, some materials that render well performances on OER catalyst also exhibit excellent EHCO activity. For instance, transition metal (especially Co and Ni) based‐LDHs materials are naturally considered to be ideal candidates for EHCO catalysts, which not only exhibit excellent performance, but also provide a chance to understand the reaction's mechanism. In this part, we focus on the development of LDHs materials toward EHCO for the conversion process of three common organics compounds in aqueous environment (Table 2). We tried our best to appraise the performances (such as overpotential and faradaic efficiency (FE)) of these electrocatalysts and raised the key issues of current researches and perspectives for EHCO.

**TABLE 2:** EHCO performances based on LDHs electrocatalysts

Organic compounds	Catalyst	Electrolyte/organics concentration	*E* [V *vs*. RHE] @ Current density	Tafel slope [mV dec^−1^]	FE [%] @ *E* [V *vs*. RHE]	Ref.
Methanol	NiCo‐LDH	1 M KOH/0.5 M	1.33 @ 10 mA cm^−2^	17	−	^[^ ^87^ ^]^
	NiCo‐LDH	1 M KOH/0.5 M	1.77 @ 761 mA cm^−2^			^[^ ^88^ ^]^
	NiAl‐LDH	1 M KOH/2 M	1.42 @ 5 mA cm^−2^	−	−	^[^ ^89^ ^]^
	NiFe‐LDH	1 M KOH/2 M	1.42 @ 20 mA cm^−2^	−	−	^[^ ^90^ ^]^
	MgAl‐LDH/Pt	0.5 M NaOH/1 M	0.78 @ 18 mA cm^−2^	−	−	^[^ ^91^ ^]^
	Co_*x*_P@NiCo‐LDH	1 M KOH/0.5 M	1.24 @ 10 mA cm^−2^	58	∼100 @ 1.35	^[^ ^92^ ^]^
	CeO_2_/NiV‐LDH	1 M KOH/0.5 M	1.62 @ 240 mA cm^−2^	−	−	^[^ ^93^ ^]^
Ethanol	Exfoliated NiAl‐LDH	1 M NaOH/1 M	1.59 @ 45.8 mA cm^−2^	−	−	^[^ ^94^ ^]^
	MgFe‐LDH	1 M NaOH/1 M	1.37 @ 1.2 mA cm^−2^	−	−	^[^ ^95^ ^]^
	NiFe‐LDH@MnO_2_	1 M KOH/1 M	1.48 @ 2.2 mA cm^−2^	−	−	^[^ ^96^ ^]^
	Pd/CoAl‐LDH	1 M NaOH/0.05 M	0.71 @ 2 mA cm^−2^	−	−	^[^ ^97^ ^]^
	NiFe‐LDHs/CNT	1 M KOH/1 M	∼1.45 @ 102 mA cm^−2^	−	−	^[^ ^98^ ^]^
Glucose	NiAl‐LDH	0.1 M NaOH/21 mM	1.74 @ 8 mA cm^−2^	−	−	^[^ ^99^ ^]^
	NiCo‐LDH	0.1 M KOH/1 M	1.50 @ 16.7 mA cm^−2^	−		^[^ ^100^ ^]^
	NiFeO*_x_* derived from NiFe‐LDH	1 M KOH/100 mM	1.30 @ 87.6 mA cm^−2^	19	87 @ 1.30	^[^ ^82^ ^]^
HMF	NiFe‐LDH	1 M KOH/10 mM	1.32 @ 20 mA cm^−2^	75	99.4 @ 1.23 77.2 @ 1.43	^[^ ^109^ ^]^
	NiFeCo‐LDH	1 M KOH/5 mM	1.53 @ 10 mA cm^−2^	68	∼90 @ 1.54	^[^ ^110^ ^]^
	NiCoMn‐LDH	1 M NaOH/1 mM	1.6 @ 50 mA cm^−2^	118	−	^[^ ^111^ ^]^
	E‐CoAl‐LDH‐NSA	1 M KOH/10 mM	1.30 @ 10 mA cm^−2^	76.67	99.4 @ 1.52 97.47 @ 1.57 69.63 @ 1.62	^[^ ^57^ ^]^
	CoFe@NiFe	1 M KOH/10 mM	1.31 @ 10 mA cm^−2^	82.8	75.7 @ 1.34 46.82 @ 1.50	^[^ ^117^ ^]^
	Cu_*x*_S@NiCo LDHs	1 M KOH/10 mM	1.34 @ 20 mA cm^−2^	59.6	∼90 @ 1.32	^[^ ^118^ ^]^
Furfural	NiCoMn‐LDH	1 M NaOH/1 mM	1.58 @ 50 mA cm^−2^	112	−	^[^ ^111^ ^]^
Urea	NiFe‐LDH	1 M KOH/0.33 M	1.36 @ 30 mA cm^−2^	33	−	^[^ ^116^ ^]^
	CoMn‐LDH	1 M KOH/0.33 M	1.326 @ 10 mA cm^−2^	73	−	^[^ ^117^ ^]^
	NiFeCo‐LDH	1 M KOH/0.33 M	1.35 @ 10 mA cm^−2^	31	−	^[^ ^118^ ^]^
	CoFeCr‐LDH	1 M KOH/0.33 M	1.305 @ 10 mA cm^−2^	85	−	^[^ ^119^ ^]^
	NiFeRh‐LDH	1 M KOH/0.33 M	1.45 @ 603 mA mg^−1^	35	−	^[^ ^120^ ^]^
	NiCo‐LDH‐NO_3_^−^	1 M KOH/0.33 M	∼1.61 @ 100 mA cm^−2^	91	88 @ 1.50	^[^ ^121^ ^]^
	NiCo‐LDH‐ [MoS_4_]^2−^	1 M KOH/0.33 M	∼1.34 @ 10 mA cm^−2^	29	−	^[^ ^122^ ^]^
	P‐NiCoZn LDH	1 M KOH/0.5 M	1.321 @ 100 mA cm^−2^	38	−	^[^ ^123^ ^]^
	FQD/CoNi‐LDH	1 M KOH/0.5 M	1.36 @ 10 mA cm^−2^	17	−	^[^ ^124^ ^]^
	NiCo‐LDH/NiCo(OH)_2_	5 M KOH/0.33 M	∼1.7 @ 390 mA cm^−2^	−	90 @ 1.70	^[^ ^125^ ^]^
	MoS_2_/Ni_3_S_2_/NiFe‐LDH	1 M KOH/0.5 M	1.396 @ 100 mA cm^−2^	36	−	^[^ ^126^ ^]^
Hydrazine	NiFe‐LDH	1 M KOH/2 M	1.31 @ 100 mA cm^−2^	−	−	^[^ ^31^ ^]^
	Ru_1_/mono‐NiFe	1 M KOH/0.2 M	1.26 @ 10 mA cm^−2^	107	∼98	^[^ ^129^ ^]^
	Rh/NiFe‐5.4	1 M KOH/0.2 M	1.38 @ 10 mA cm^−2^	181	−	^[^ ^130^ ^]^
	S‐CuNiCo‐LDH	0.1 M KOH/20 mM	1.0 @ 185.1 mA cm^−2^	73.3	−	^[^ ^131^ ^]^
	Ni/NiFe‐LDH	3 M KOH/0.1 M	0.2 @ 157 mA cm^−2^	83	−	^[^ ^132^ ^]^
	Cu_1_Ni_2_‐N derived from CuNi‐LDH	1 M KOH/0.5 M	0.096 @ 50 mA cm^−2^	44.1	−	^[^ ^134^ ^]^
	CoNi‐R‐S derived from CoNi‐LDH	0.1 M KOH/2 M	1.0 @ 117.8 mA cm^–2^	67	−	^[^ ^135^ ^]^
Diphenyl sulfide	CoFe‐LDH	MeCN/H_2_O/0.5 mM	1.39 @ 5 mA cm^–2^	−	−	^[^ ^138^ ^]^
1‐Phenylethanol	CoMn‐LDH	1 M KOH/0.1 M	1.36 @ 10 mA cm^–2^	−	−	^[^ ^139^ ^]^

As mentioned above, the upgrading of alcohols (such as ethanol, benzyl alcohol, and glycerol) into valuable products has been considered as the promising alternative reaction of OER at anode. LDHs‐based electrocatalyst has been widely used in the oxidation of alcohol in alkaline environment due to their facile preparation and high activity in EHCO.^[^ ^82^, ^87^, ^88^, ^89^, ^90^, ^91^, ^92^, ^93^, ^94^, ^95^, ^96^, ^97^, ^98^, ^99^, ^100^ ^]^ Benefits from their architecture flexibility, varied LDHs with highly exposed active sites, and high surface area can be obtained, leading to fast mass and electron transfer, which result in the significantly enhanced activity toward alcohol oxidation. For instance, our group reported a hierarchical MgFe‐LDH microsphere with adjustable internal structure via a facile surfactant‐templated strategy for highly efficient ethanol oxidation.^[^ ^95^ ^]^ The hollow microsphere structure provided large surface area and suitable mesopore structure, which favor the mass transport of electrolytes and thus improve corresponding the faradaic redox reaction. Moreover, LDHs were also reported to combine with noble metal catalyst (e.g., Au, Pt, and Pd) to enhance their alcohol oxidation performances to realize the application in fuel cells.^[^ ^91^, ^97^, ^101^ ^]^ The introduction of noble metal nanoparticles can effectively enhance the intrinsic activity of LDHs toward alcohol activity.^[^ ^97^ ^]^ Additionally, LDHs can increase the concentration of OH^−^ around noble metal sites, thus removing the CO (main reason for the poisoning of noble metal in practical use) adsorbed on the surface of precious metals.^[^ ^101^ ^]^ The high electroconductivity and high tolerance to the poisoning intermediate of LDHs/noble metal hybrids contribute to the enhanced activity and durability toward alcohol oxidation.^[^ ^102^ ^]^ It is worth mentioning that the alcohol oxidation can also be coupled with other reduction reactions, such as CO_2_ reduction reaction as well as oxygen reduction reaction. For instance, Shi and co‐workers reported CO_2_ reduction reaction coupled with methanol oxidation to realize efficient formic acid production at both anode and cathode.^[^ ^103^ ^]^ The replacement of OER at anode with methanol oxidation significantly lowers the energy input of overall formic acid production with the cell voltage of only 0.93 V to reach 10 mA cm^–2^.

For the oxidation reactions of alcohols with larger molecules, LDHs‐based catalysts can also exhibit great catalytic activity. Based on the unique layer structure of LDHs, our group fabricated ultrathin films based on CoFe‐LDH and manganese porphyrin (Mn–TPPS) via a layer‐by‐layer assembly strategy for electrochemical glucose oxidation, which can be used as a good sensor.^[^ ^104^ ^]^ The as‐prepared CoFe‐LDH/Mn–TPPS_6_ exhibited a lower electron transfer resistance of ∼100 Ω (∼450 Ω for pristine CoFe‐LDH), resulting in highly enhanced electrocatalytic activity toward glucose oxidation. Recently, a NiFe oxide (NiFeO*_x_*) and a nitride (NiFeN*_x_*) electrode derived from NiFe‐LDH nanosheets array have been reported with high activity and selectivity toward glucose oxidation due to the high exposed active sites and low charge transfer resistance.^[^ ^82^ ^]^ Through the replacement of OER with glucose oxidation, the potential of two electrode cells to reach 200 mA cm^−2^ decreased significantly from 1.73 to 1.48 V. Meanwhile, the cost of glucaric acid production through this electrochemical strategy is calculated to be $9.32 kg^−1^, much lower than that of non‐electrochemical method ($17.04 kg^−1^).

As the renewable and available green organic carbon resource, biomass is considered to be an ideal alternative to fossil resource for the manufacture of liquid fuels and value‐added chemicals. Among them, furfural compounds are gaining increasing attention and are accepted as important platform molecules due to their broad industrial application.^[^ ^105^, ^106^, ^107^, ^108^ ^]^ Especially, 5‐hydroxymethylfurfural (HMF) is the most researched “star” molecule due to its facile conversion into a variety of block compounds for chemical industry. The full oxidation of HMF at both sides on the furan ring can produce 2,5‐furandicarboxylic acid (FDCA), which can further polymerize with ethylene glycol to form eco‐friendly plastic (polyethylene furandicarboxylate).^[^ ^59^, ^85^ ^]^ LDHs‐based materials have been reported as competitive electrocatalyst toward the oxidation of HMF into FDCA with high FE and selectivity, such as NiFe‐LDH, NiFeCo‐LDH, and NiCoMn‐LDH.^[^ ^109^, ^110^, ^111^ ^]^ Among them, NiFe‐LDH was firstly reported to boost an efficient HMF oxidation reaction (HMFOR) with a low onset potential of 1.25 V *vs*. RHE and a high FE of 99.4% under the potential of 1.23 V *vs*. RHE.^[^ ^109^ ^]^ However, these catalysts just can maintain high efficiency for HMFOR only at lower voltages (<1.35 V *vs*. RHE), meaning that the OER process still dominates at higher voltages. This greatly limits the rate of FDCA production and corresponding hydrogen production at practical applications. Therefore, high current density combined with high FE toward FDCA is the key to give efficient FDCA production coupled with hydrogen production. To achieve this goal, our group explored the idea of boosting HMFOR while inhibiting OER through tuning the surface structure and electronic environment of LDHs electrocatalysts.^[^ ^59^ ^]^ Typically, the as‐synthesized vacancies‐rich CoAl‐LDH NSA displays remarkable performance for HMFOR with a FE over 95% toward FDCA in a wide potential range of 1.37−1.57 V *vs*. RHE, which stands at the highest level compared to previous electrocatalysts. Furthermore, the powder products of FDCA were obtained with the purity of 99.45%, which truly realized the production of value‐added organics. Combining with theoretical calculations, it is found that the introduction of oxygen vacancies can enhance the adsorption of HMF while reducing the energy barrier of the oxidation rate‐determining step. Moreover, the *in‐situ* FT‐IR further illustrates the introduction of oxygen vacancies results in a significantly enhanced adsorption of C═O bond of aldehyde group and C═C bond on the furan ring of HMF. In other reports, the *in‐situ* FT‐IR, Raman, and sum frequency generation vibrational spectroscopy have also been used to illustrate the generation of intermediates and final products based on the changes of functional groups in the HMF oxidation process.^[^ ^81^, ^112^, ^113^ ^]^

Apart from alcohol molecules, N‐based compounds have also been regarded as the candidates for fuel cells such as urea and hydrazine.^[^ ^86^ ^]^ Recently, the oxidation of amino‐containing compounds has been widely used as the anode reaction for electrochemical hydrogen production owing to their thermodynamic lower potentials. Taking the urea oxidation reaction (CO(NH_2_)_2_ + H_2_O → CO_2_ + N_2_ + 3H_2_) as an example, the standard Gibbs free energy of the reaction is 36.36 kJ mol^–1^, much lower than that of overall water splitting (237.101 kJ mol^–1^).^[^ ^3^ ^]^ In addition, the widely available urea is a major pollutant causing eutrophication in water resources. Thus, it is promising to substitute OER by using urea oxidation to simultaneously reduce the overpotential of anode and achieve the value‐added reactions. Ni‐based LDHs have been reported to display superior urea oxidation activity than noble metals (Pt, Pt–Ir, Rh) in alkaline media.^[^ ^114^, ^115^, ^116^, ^117^, ^118^, ^119^, ^120^, ^121^, ^122^, ^123^, ^124^, ^125^, ^126^ ^]^ Moreover, the compositional tunability for both laminate cation and interlayer anion of LDHs can be further exploited to improve their urea oxidation performance. It has been reported that the doping of Co cations optimized the electroconductivity and regulated the electronic structure of NiFeCo‐LDH, leading to a highly enhanced urea oxidation performance with lower onset potential (0.28 V *vs*. SCE to reach 10 mA cm^–2^) than NiFe‐LDH (0.38 V) in 1 M KOH with 0.33 M urea.^[^ ^114^ ^]^ Meanwhile, introducing [MoS_4_]^2–^ into the interlayer space was investigated to be effective on tuning the electronic structure of NiCo‐LDH, which facilitated the formation of active MOOH species for urea oxidation.^[^ ^116^ ^]^ As a result, the [MoS_4_]^2–^‐intercalated NiCo‐LDH exhibited a superior urea oxidation performance, which required only ∼1.34 V *vs*. RHE to reach 10 mA cm^–2^ in 1 M KOH + 0.33 M urea solution with outstanding stability over 24 h. In the study of urea oxidation, the *in‐situ* generated hydroxyl oxide species under oxidation potentials in alkaline solutions are widely considered to be the active species in small molecule oxidation reactions, which can be identified by means of characterization such as *in‐situ* Raman or *in‐situ* X‐ray photoelectron spectroscopy.^[^ ^127^, ^128^ ^]^

Compared to the urea oxidation, the oxidation of hydrazine can be performed at lower standard voltage (−0.33 V *vs*. RHE, while 0.37 V *vs*. RHE for urea oxidation). Besides, the products of hydrazine oxidation are nitrogen and water with no greenhouse gases emission. Therefore, the oxidation of hydrazine is an ideal candidate for anode reaction to be coupled with hydrogen production. Transition metal LDHs, as a typical kind of non‐noble metal catalyst are widely used in hydrazine oxidation in alkaline media.^[^ ^31^, ^129^, ^130^, ^131^, ^132^, ^133^, ^134^, ^135^ ^]^ Our group fabricated a series of Fe‐containing LDHs via an electrosynthesis strategy,^[^ ^31^ ^]^ where the NiFe‐LDH exhibited excellent activity toward hydrazine oxidation with a low potential of 244 mV *vs*. SCE at 100 mA cm^–2^. The 3D nano‐array structure provided abundant reaction sites and the conductive substrate provided low charge transfer resistance for hydrazine oxidation. To realize the further enhancement of hydrazine oxidation activity, Ru single atoms were decorated on mono‐layer NiFe‐LDH by Song et al.^[^ ^129^ ^]^ Theoretical calculation demonstrated the atomic dispersed Ru sites on LDHs can steady the intermediates (*N_2_H_3_ and *N_2_H) in hydrazine oxidation process and decrease the band gap energy and reaction barrier. Typically, the Ru/mono‐NiFe‐LDH with Ru atom loading amount of 1.6% demonstrated the best hydrazine oxidation activity with only 1.26 V *vs*. RHE to reach 10 mA cm^–2^ and a FE of ∼98%. Besides, transition metal sulfides (TMSs) and transition metal nitrides (TMNs) derived from LDHs have also been reported as high‐efficient electrodes for hydrazine oxidation. For instance, we fabricated hierarchical CoNi‐sulfides nanoarrays (denoted as CoNi‐R‐S) via an *in‐situ* reduction of CoNi‐LDH followed by a sulfidation process for high‐performance hydrazine oxidation.^[^ ^135^ ^]^ Theoretical calculations reveal that CoNi‐sulfide shell significantly decreases the activation energy of hydrogen dissociation. Meanwhile, the core‐shell structure of CoNi‐sulfide provides highly conductive pathway for electron transportation. Thus, the as‐obtained CoNi‐R‐S catalyst presented a current of 117.8 mA cm^–2^ at 1.0 V *vs*. RHE in 0.1 M KOH containing 2 M hydrazine, which was 5 times higher than that of CoNi‐LDH (23.7 mA cm ^–2^). Meanwhile, Wang et al. fabricated Cu‐based TMNs electrocatalyst for HzOR through constructing CuNi‐LDH nanosheets on carbon fiber cloth, followed by a thermal ammonolysis process (denoted as Cu_1_Ni_2_‐N).^[^ ^134^ ^]^ Due to the porosity and high conductivity, the as‐prepared Cu_1_Ni_2_‐N electrocatalyst exhibited high hydrazine oxidation activity with the potential of only 0.5 and 96.9 mV to reach the current of 10 and 50 mA cm^–2^ respectively, much lower than that of CoNi‐LDH. A two‐electrode electrolyzer with the Cu_1_Ni_2_‐N simultaneously served as cathode and anode delivered an extremely low voltage of 0.24 V to reach 10 mA cm^−2^ in the aqueous solution of 1 M KOH with 0.5 M hydrazine, while a rather high voltage of 1.63 V is still needed in OER/HER coupled water splitting system. Interestingly, the hydrazine oxidation has also been reported to be coupled with oxygen reduction reaction recently, which achieves self‐powered hydrogen production.^[^ ^136^, ^137^ ^]^ Among them, Qiu et al. developed a hydrogen production system operating under seawater environment. In this work, the generation of chlorine at anode side was completely avoided and the hydrogen production was accelerated simultaneously when OER was replaced by hydrazine oxidation. Moreover, they coupled hydrazine oxidation with oxygen reduction to form a direct hydrazine fuel cell, which can be further assembled into an above‐mentioned hybrid seawater electrolyzer to drive the self‐powered hydrogen production.^[^ ^137^ ^]^

Recently, LDHs‐based catalysts were reported to be used in more complex organic molecules upgrading, which further expanded the utilization of LDHs in petrochemical industry and biomass valorization. For instance, CoFe‐LDH has been found with high performance for electrochemical selective oxidation of sulfides (including complex compounds like ricobendazole, omeprazole, sulindac, and amino acid methionine) to sulfoxides.^[^ ^138^ ^]^ Significantly, it realized the gram‐scale synthesis of diphenyl sulfoxide and amino acid methionine with the yield of 86% and 83%, respectively. Other than that, manganese‐doped cobalt oxyhydroxide (MnCoOOH) catalyst derived by *in‐situ* electrochemical activation of CoMn‐LDH was reported to upgrade lignin derivatives into carboxylates through electrochemical oxidative C(OH)−C bonds cleavage.^[^ ^139^ ^]^ In this work, lignin models with different functional groups are convergently transformed into benzoate over MnCoOOH at anode side with a high yield of 91.5%. Meanwhile, the hydrogen production at cathode side was simultaneously increased by 14 folds. Theoretical calculations confirmed that the rate‐determining step of lignin oxidation and the main reactive oxygen species. Moreover, the doping of high valance Mn species enhances the adsorption of OH^–^ and accelerates the formation of main active species (OH^*^) toward C(OH)−C bonds cleavage. Very recently, a nickel‐modified cobalt phosphide (CoNiP) derived from CoNi‐LDH was reported as an efficient electrocatalyst for electrocatalytic upcycling of polyethylene terephthalate (PET) plastic to valuable commodity chemicals (potassium diformate and terephthalic acid) and H_2_ fuel, showing the potential to implement waste PET upcycling to value‐added products in a sustainable way.^[^ ^140^ ^]^

## 4 SUMMARY AND PERSPECTIVE

**FIGURE 4:**
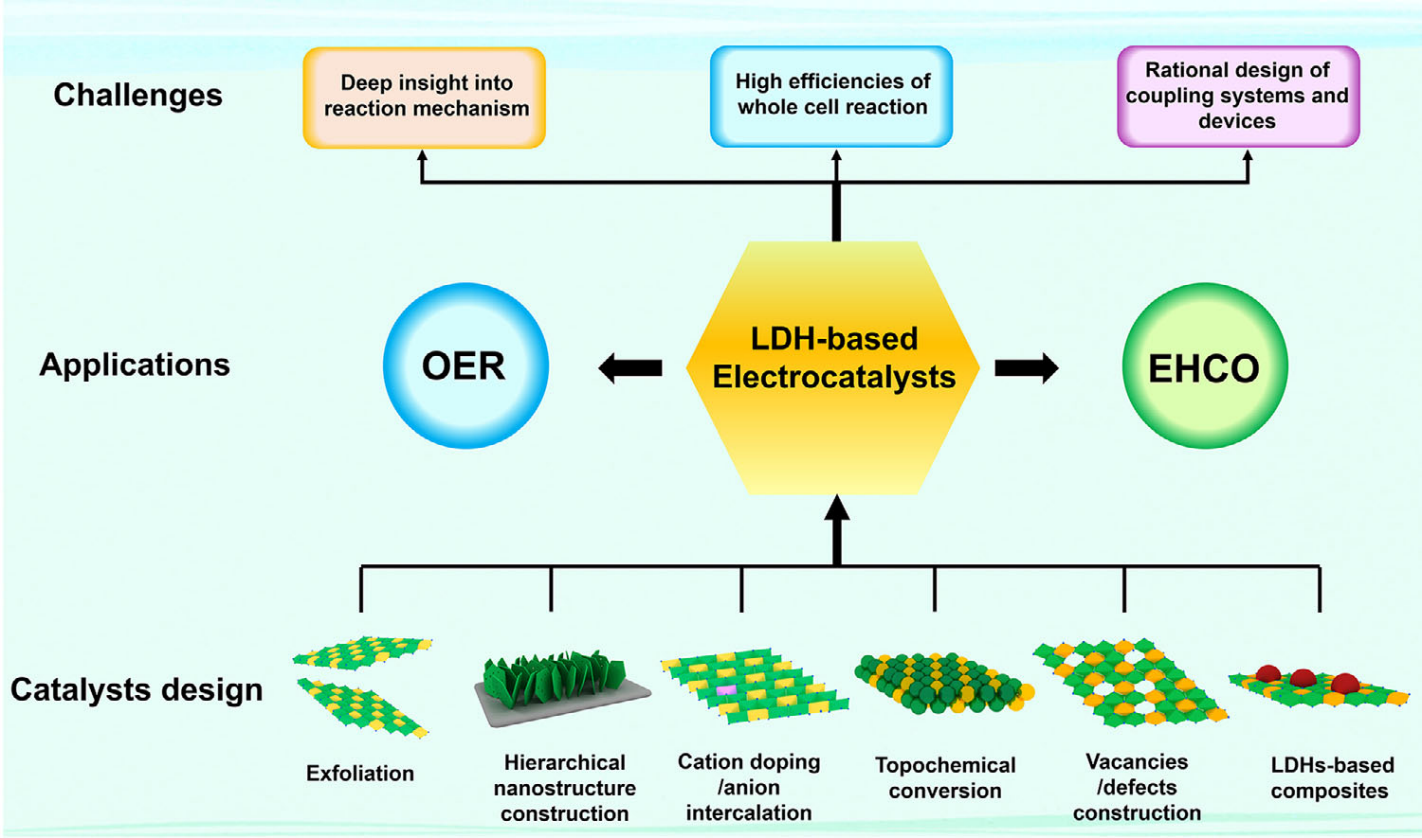
Catalysts design, application, and challenges of LDH‐based electrocatalysts for OER and EHCO

Electrochemical water splitting has been regarded as one of the most green and promising ways for hydrogen production. How to improve the efficiencies of electrolysis is the key to decrease the cost of hydrogen production. In this review, we summarized two strategies to increase the economic benefits of electrochemical water splitting: the development of advanced OER catalysts and replacing OER with EHCO. Through these two strategies, the overpotential of water splitting was significantly reduced, leading to lower cell voltage and energy consumption of hydrogen production. Moreover, suitable design of anode reaction can realize the production of higher value‐added products, further improving the economic benefits of hydrogen production from electrochemical water splitting. With regard to the strategy of EHCO, we have classified the most reported oxidation reactions of organic compounds into three categories (oxidation of alcohol, furfural compounds, and amino‐containing compounds) by using LDHs‐based electrocatalysts. Although these progresses, it is still a tough journey to realize the practical applications of hydrogen production via electrochemical water splitting with much reduced cost. Here, we present several major challenges in this field and look forward to the corresponding development directions and approaches (Figure 4).

### 4.1 Deep insight into reaction mechanism

In recent years, with the advancement of characterization techniques and research, the OER process and mechanism involved in LDHs‐based catalysts have been widely studied and reported. By contrast, there is a lack of research on the process and mechanism of electrocatalytic oxidation of other molecules, especially the organic molecules. The fine structure and even the phase of some electrocatalysts can be easily changed under the actual reaction environment. This phenomenon is usually called catalyst reconstruction and has been widely investigated in the terms of OER. For instance, transition metal‐based sulfides and phosphides have been reported to be transformed into corresponding hydroxides in alkaline media and further turned into oxyhydroxide under the applied oxidation potential. However, monitoring of the reconstitution process and recognition of the actual active sites for organics oxidation electrocatalysts was barely discussed in previous reports. Yet it is crucial for the revelation of reaction mechanism and the design of catalysts. The *in‐situ* techniques provide a big chance to reveal the chemical species formed on the surface of the electrode as well as to monitor the changes of structure and valance of catalysts, such as *in‐situ* Raman, XPS, and XAS. But, it still remains a challenge to reveal the nature of electrocatalysts and related reaction mechanisms involved in the complex organic oxidation reactions.

### 4.2 High efficiencies of whole cell reaction

Most organic oxidation catalysts are simultaneously excellent OER catalysts, which leads to competition between OER and organic oxidation reactions. This leads to the catalysts just can maintain high efficiency for organic oxidation at lower voltages, which seriously limits the rate of the production of value‐added products and corresponding hydrogen production at cathode. To address this issue, some considerations have been proposed as follows: (1) Exploration of non‐OER active materials as efficient catalysts for organic oxidation reactions. Primarily, a solid understanding of the thermodynamics and kinetics of the oxidation reactions is necessary, which can help us to select the suitable alternative oxidation reactions (with lower theoretical potentials compared to OER). Moreover, based on the clarification of the mechanism of specific oxidation reaction and OER, we could avoid the coupling of oxygen atoms on the catalyst surface via rational catalysts design, thereby inhibiting OER and widening the potential window for organic oxidation. (2) With the regard to the poorly soluble organics oxidation in aqueous environment, hydrophobic modification of electrodes could be a strategy to inhibit OER. (3) Enhance the selectivity toward organics oxidation by changing the nature of electrolyte. For instance, decreasing the concentration of OH^–^ and increasing the concentration of organic substrate in a suitable range could effectively slow down the OER process and enhance the efficiency of organic oxidation. Besides, the addition of other electrolyte molecules also has the potential to modulate the kinetic rate of OER and the oxidation of organics.

### 4.3 Rational design of coupling systems and devices

Current researches have focused on the construction of high‐performance catalysts, while the design of entire coupling device and system is also necessary. Although some anode reactions are effective in reducing the overall cell voltage, the value of the product obtained from the anode is usually not so high. Selecting a suitable oxidation reaction is a feasible strategy to lower the energy consumption and enhance the benefits for electrochemical hydrogen production. The anodic substrate concentration in the current studies tends to be low (usually lower than 10 mM). However, the actual production process needs to be carried out under high substrate concentration conditions, which makes it desirable to construct electrodes that are efficient and stable under high substrates concentrations. Additionally, the anodic products have been reported to remain in the electrolyte, which have not been separated and purified. To ensure the efficient production of hydrogen and high value‐added products, the corresponding separation and purification units are necessary to be equipped to obtain the products.

Therefore, future research is still highly needed that not only focus on the design of catalysts with high performance, but also on the deeper insight into the reaction mechanism and the design of new reaction systems and devices to achieve future applications. We hope this review can provide inspiration for the design of advanced systems and electrocatalysts to promote the development and application of electrochemical hydrogen production technology.

## CONFLICT OF INTEREST

The authors declare no conflict of financial interest.
